# Hyperglycemia induces differential change in oxidative stress at gene expression and functional levels in HUVEC and HMVEC

**DOI:** 10.1186/1475-2840-12-142

**Published:** 2013-10-05

**Authors:** Hemang Patel, Juan Chen, Kumuda C Das, Mahendra Kavdia

**Affiliations:** 1 Department of Biomedical Engineering, Wayne State University, 2322 Engineering, 5050 Anthony Wayne Dr., Detroit, MI 48202, USA; 2 Department of Anesthesiology, Texas Tech University Health Sciences Center, Lubbock, TX 79430, USA

**Keywords:** Endothelial dysfunction, Microvascular dysfunction, Systems biology, Oxidative stress, Hyperglycemia, HUVEC, HMVEC, Vascular metabolic memory

## Abstract

**Background:**

Endothelial dysfunction precedes pathogenesis of vascular complications in diabetes. In recent years, the mechanisms of endothelial dysfunction were investigated to outline strategies for its treatment. However, the therapies for dysfunctional endothelium resulted in multiple clinical trial failures and remain elusive. There is a need for defining hyperglycemia-induced endothelial dysfunction with both generic and specific dysfunctional changes in endothelial cells (EC) using a systems approach. In this study, we investigated hyperglycemia-induced endothelial dysfunction in HUVEC and HMVEC. We investigated hyperglycemia-induced functional changes (superoxide (O_2_‾), and hydrogen peroxide (H_2_O_2_) production and mitochondrial membrane polarization) and gene expression fingerprints of related enzymes (nitric oxide synthase, NAD(P)H oxidase, and reactive oxygen species (ROS) neutralizing enzymes) in both ECs.

**Method:**

Gene expression of NOS2, NOS3, NOX4, CYBA, UCP1, CAT, TXNRD1, TXNRD2, GPX1, NOX1, SOD1, SOD2, PRDX1, 18s, and RPLP0 were measured using real-time PCR. O_2_‾ production was measured with dihydroethidium (DHE) fluorescence measurement. H_2_O_2_ production was measured using Amplex Red assay. Mitochondrial membrane polarization was measured using JC-10 based fluorescence measurement.

**Results:**

We showed that the O_2_‾ levels increased similarly in both ECs with hyperglycemia. However, these endothelial cells showed significantly different underlying gene expression profile, H_2_O_2_ production and mitochondrial membrane polarization. In HUVEC, hyperglycemia increased H_2_O_2_ production, and hyperpolarized mitochondrial membrane. ROS neutralizing enzymes SOD2 and CAT gene expression were downregulated. In contrast, there was an upregulation of nitric oxide synthase and NAD(P)H oxidase and a depolarization of mitochondrial membrane in HMVEC. In addition, ROS neutralizing enzymes SOD1, GPX1, TXNRD1 and TXNRD2 gene expression were significantly upregulated in high glucose treated HMVEC.

**Conclusion:**

Our findings highlighted a unique framework for hyperglycemia-induced endothelial dysfunction. We showed that multiple pathways are differentially affected in these endothelial cells in hyperglycemia. High occurrences of gene expression changes in HMVEC in this study supports the hypothesis that microvasculature precedes macrovasculature in epigenetic regulation forming vascular metabolic memory. Identifying genomic phenotype and corresponding functional changes in hyperglycemic endothelial dysfunction will provide a suitable systems biology approach for understanding underlying mechanisms and possible effective therapeutic intervention.

## Background

Diabetes, a complex metabolic syndrome, is a rapidly growing public health burden in both developed and developing countries. Among all pathophysiologies associated with diabetes, micro and macrovascular complications are implicated in most conditions leading to morbidity and mortality in diabetic patients [1]. Hyperglycemic condition associated with diabetes dysregulates endothelial function that leads to initiation and propagation of vascular complications and dysfunction [2,3]. The understanding and amelioration of endothelial dysfunction is important for diabetic vascular complications.

The onset of endothelial dysfunction begins with disruption of balance amongst vasorelaxation and vasoconstriction factors. Under hyperglycemic condition, an increase in intracellular reactive oxygen species (ROS) is responsible for pathophysiological changes including nitric oxide (NO) synthesis inhibition, vascular inflammation, insulin resistance, neovascularization, leukocyte adhesion, and protein and macromolecule glycation [4-6]. Pharmacological therapies including antioxidants, vitamin E, L-arginine, calcium antagonists, β-blockers, renin-angiotensin system inhibitors, statins, insulin-resistance improving drugs, erythropoietin, and tetrahydrobiopterin have been shown to ameliorate endothelial dysfunction [2,5,7-9]. However, their efficacy on treating dysfunctional endothelium varies with different disorders and in different parts of vasculature [2,5,7-9]. Several of the clinical trials with antioxidants have failed to show benefits even though in vitro and animal studies have shown significant improvement [6,9]. Our understanding of the mechanisms of hyperglycemia-induced oxidative stress and resulting endothelial cell dysfunction from a systems perspective is lacking. While the reason for justifying differential efficacies of therapeutic strategies remains unclear, these findings have raised the need for improving the understanding for hyperglycemia-induced pathogenesis of endothelial dysfunction in different parts of vasculature.

In normal physiology, endothelial cells (EC) regulate vascular homeostasis through NO production and its bioavailability [10]. Even though critical for wide ranges of cell signaling and cell-cell communication processes, NO is susceptible to inactivation through intracellular superoxide (O_2_‾) [10]. In hyperglycemia, intracellular O_2_‾ increases from sources including NAD(P)H oxidase family enzymes, xanthine oxidase, cyclooxygenase, uncoupled constitutive nitric oxide synthase (eNOS), mitochondrial electron transport, glucose oxidase, and lipooxygenase [6,11-14]. Intracellular O_2_‾ is a relatively short-lived species, which can get dismutated by superoxide dismutase (SOD) enzyme and self-dismutation to hydrogen peroxide (H_2_O_2_) in addition to its rapid reaction with NO.

Unlike O_2_‾, H_2_O_2_ is more stable ROS [15]. High glucose exposure increases H_2_O_2_, which is a result of rapid dismutation of O_2_‾ in mitochondria and an increase in NAD(P)H oxidase-4 (NOX4) activity in cytosol [16,17]. The lower level of H_2_O_2_ causes vasorelaxation along with induction and activation of nitric oxide synthase (NOS) [15,18], whereas the higher level of H_2_O_2_ promote vasoconstriction and causes oxidative damage to the vasculature [18]. During oxidative stress condition, the intracellular H_2_O_2_ levels are tightly regulated through i) a direct involvement of catalase, peroxiredoxin and thioredoxin enzyme networks, and ii) an indirect involvement from uncoupling proteins, and Nrf-2 expression [19,20]. Therefore, endothelial function is regulated though a complex network of regulation in NO production, O_2_‾ production and dismutation, and peroxide clearance.

For a generic understanding of hyperglycemia-mediated oxidative stress and endothelial dysfunction, the current trend in literature emphasizes the studying and understanding of individual sources of endothelial dysfunction in a specific type of endothelial cells or vasculature segment. In normal physiology, endothelial cells originated from different organs and parts of vasculature express different functional characteristics that are based on endothelial function, gene expression, and proteomic profile at homeostasis [21-23]. Such functional differences in endothelial cells can be precursors of their variable behavior under both physiological and pathophysiological conditions [24].

Hyperglycemia has been shown to increase O_2_‾ production and induce endothelial dysfunction. However, the production and involvement of O_2_‾ sources varies across ECs from different parts of vasculature [25-28]. In human glomerular endothelial cells (HGEC) and human aortic endothelial cells (HAEC), the major source of hyperglycemia-induced oxidative stress and O_2_‾ production is uncoupled eNOS and NAD(P)H oxidase, respectively [26,28]. Hyperglycemia-induced endothelial dysfunction is also differentially regulated in ECs from different part of the vasculature. Karabach et al. [29] showed that HUVEC and EA.hy.926 have significantly different cell viability and O_2_‾ formation in hyperglycemia. Pala et al. [30] showed that HAEC and HMVEC have differential dipeptidyl peptidase-4 expression and activity levels in hyperglycemia. Wang et al. [24] showed that endothelial cells derived from myocardial microvasculature and aorta of type 2 diabetic rats differentially expressed growth factors and impaired angiogenesis only in myocardial microvascular endothelial cells.

These studies show a need for the understanding of mechanisms for the differences in hyperglycemia-induced endothelial cell functional changes across the vasculature. Further studying ECs from different parts of the vasculature will enhance the system wide understanding for hyperglycemia-induced vascular dysfunction [31]. In this study, we investigated hyperglycemia-induced endothelial dysfunction in HUVEC and HMVEC. To compare changes in functional behavior of both cell types, levels of O_2_‾, H_2_O_2_, and mitochondrial membrane polarization were measured following 24 hours of 25 mM D-glucose exposure. In addition, we measured gene expression changes important to these functional changes including nitric oxide synthase enzymes (NOS2 and NOS3), NAD(P)H oxidase enzymes (NOX1, NOX4, and CYBA), ROS neutralizing enzymes such as superoxide dismutase (SOD1 and SOD2), catalase (CAT), peroxiredoxin (PRDX1), glutathione peroxidase 1 (GPX1) and thioredoxin reductase 1 and 2 (TXNRD1 and TXNRD2), oxidative stress activated transcription factor, Nrf-2, (NFE2L2), and uncoupling protein 1(UCP1). Findings from this study outline a relationship between functional changes and the underlying genomic phenotype to improve understanding of differential effects of hyperglycemia on endothelial cells.

## Methods

### Cell culture and experimental protocol

Primary HUVEC and HMVEC were purchased from Lonza, MD. HUVEC and HMVEC were grown using EGM-2 media supplemented with Bullet kit (Lonza, MD). Media was changed every 48 hours until cells reached 80 to 90% confluency. At confluency, cells were either subcultured or used for experiments. For all experiments, cells growing at passage either 4 or 5 were used and experiments were carried out using M199 media supplemented with low serum growth supplement kit (Life Tech, CA). This media was also used as control and had 5.6 mM D-glucose. For high glucose treatment, D-glucose was added to M199 for a final concentration of 25 mM (HG-M199). For experiments, HUVEC and HMVEC were seeded into separate 6 well plates. After 24 hours of seeding, half of the sample wells in 6 well plates were treated with HG-M199 media, and the other half were treated with the control media.

### O_2_‾ measurement using DHE fluorescence

Dihydroethidium (DHE), was used to measure O_2_‾ in HUVEC and HMVEC following 24 hours of treatment with high glucose. At the end of the treatment period, cells were washed and incubated with 5 μM DHE in phenol red-free treatment medium for 1 hour. During the incubation period cells, we measured 2-hydroxyethidium (2*-*OH*-*E^+^), a specific product from DHE and O_2_‾ interaction, intensity using a microplate reader. Excitation (Ex) of 508 ± 10 nm and emission (Em) of 560 ± 20 nm were used for a specific measurement of 2*-*OH*-*E^+^[32-34]. Alternatively, fluorescence microscopy was also used to capture differences in fluorescence intensities at the end of 1 hour incubation with DHE.

### H_2_O_2_ measurement

After treating cells, media supernatants were collected to measure H_2_O_2_ using Amplex® Red based assay (Life Tech, CA). In brief, at the time interval of 3, 6, 9, and 24 hours media supernatants were collected and stored in aliquots at -80°C and different wells were used for each time point. Supernatants were quickly thawed and centrifuged immediately before use. As per manufacturer’s instruction, a lower H_2_O_2_ concentration range was used for a standard curve to quantify H_2_O_2_ in supernatants.

### Mitochondrial membrane polarization measurement

JC-10 (Enzo Life Sciences, NY) was used to measure mitochondrial membrane polarization in cells. JC-10 is a cationic fluorophore, which is rapidly taken up by cells and mitochondria due to their negative charge. Inside mitochondria, JC-10 forms J-aggregates which emit fluorescence at 590 nm (red fluorescence). Remaining JC-10 in cytosol maintains monomeric form and emits fluorescence at 525 nm (green fluorescence). Uptake levels of JC-10 in mitochondria depend on polarization state of mitochondrial membrane. Hyperpolarized mitochondria have higher uptake of JC-10 compared to depolarized. Following the treatment, cells were washed twice with PBS and then incubated with 1 μM JC-10 solution prepared in PR-free M199 media for 15 minutes. After 15 minutes of incubation, cells were incubated with 1 μg/ml Hoescht 33342 (Life technologies, CA) for 10 minutes and then quickly washed with PBS prior to fluorescence imaging and microplate reader based measurements. Microplate reader based measurement for J-monomers and J-aggregates were carried out at 485 ± 10 nm (Ex)/ 516 ± 10 nm (Em) and 528 ± 10 nm (Ex)/ 590 nm ± 10 (Em), respectively.

### RNA extraction and reverse transcription

We used RNeasy mini kit (Qiagen, CA) to extract RNA. During RNA extraction genomic DNA was also removed by DNase I digest treatment. Quantity and purity of extracted RNA samples were analyzed using spectrophotometry prior to storing them in aliquots at -80°C. All RNA samples were also analyzed for integrity and genomic DNA contamination using flash gel (Lonza, MD) prior to their use in reverse transcription. Reverse transcription reactions were performed using a high capacity cDNA reverse transcription kit (Life Technologies, CA). As per manufacturer’s protocol, 1.5 μg of extracted RNA from each treatment and control samples were converted to cDNA in a 20 μl reverse transcription reaction. Following reverse transcription, all cDNA samples were stored in aliquots at -20°C until analyzed.

### Gene expression analysis

Real-time PCR was used to check gene expression levels of NOS2, NOS3, NOX4, CYBA, UCP1, CAT, TXNRD1, TXNRD2, GPX1, NOX1, SOD1, SOD2, PRDX1, 18s, and RPLP0. Synthesized cDNA from treatment and control groups specific to both HUVEC and HMVEC were used to setup real time-PCR reactions. Gene expression levels of NOS2, NOS3, NOX4, CYBA, 18s and RPLP0 were measured using Taqman gene expression assays (Life Technologies, CA). The rest of the gene expression targets were measured using custom designed primers and SYBR green chemistry. NCBI’s Primer-BLAST feature was used to design primer sequences specific to UCP1: 5′-GCTCCAGGTCCAAGGTGAAT-3′ and 5′-ACAGCGGTGATTGTTCCCAG-3′; CAT: 5′-AGGGGCCTTTGGCTACTTTG-3′ and 5′-ACCCGATTCTCCAGCAACAG-3′; TXNRD1: 5′-GGAACTAGATGGGGTCTCGG-3′ and 5′-TCTTGCAGGGCTTGTCCTAA-3′; TXNRD2: 5′-GGTGGACTACGTGGAACCTT-3′ and 5′-TCTGCCATCTTCCTCCAGTCA-3′; GPX1: 5′-CCGGGACTACACCCAGATGA-3′ and 5′-CGTTCTCCTGATGCCCAAAC-3′; PRDX1: 5′-TCCTTTGGTATCAGACCCGA-3′ and 5′-TAAAAAGGCCCCTGAACGAG-3′; NOX1: 5′-ATCCCCCTGAGTCTTGGAAGT-3′ and 5′-CACTTCCATGCTGAAGCCAC-3′; SOD1: 5′-AGCATTAAAGGACTGACTGAAGG-3′ and 5′-GTCTCCAACATGCCTCTCTTC-3′; and SOD2: 5′-GTTGGGGTTGGCTTGGTTTC-3′ and 5′- ATAAGGCCTGTTGTTCCTTGC-3′.

All Taqman gene expression assays were setup using Taqman gene expression mastermix (Life Technologies, CA), and 75 ng of cDNA, from respective samples in 20 μl PCR reactions. Using StepOne Plus System (Life Technologies, CA) real-time PCR run was setup as follows: 2 minutes at 50°C and then 10 minutes at 95°C followed by 36 cycles of 15 seconds at 95°C, 1 minute at 60°C. Rest of the gene expression targets were measured in real-time PCR using SYBR green based detection. All SYBR green based PCR reactions were setup using 200 nM of forward and reverse primers, 56.25 ng of cDNA, and Fast SYBR green expression master mix (Life Technologies, CA) in 20 μl volume. Real-time PCR reaction compiling 20 seconds at 95°C followed by 36 cycles of 3 seconds at 95°C, 20 seconds at annealing temperature of respective primer set, and 20 seconds at 60°C steps was used to carry out the reaction. Following each SYBR green based PCR, melt curve analysis and agarose gel based quality check were performed to evaluate the quality and specificity of PCR amplifications. Furthermore, no reverse transcription, and no template controls were also ran for evaluating specificity of PCR amplification.

The cycle threshold (C_T_) values form all real-time PCR experiments were analyzed using ∆∆C_T_ method. Housekeeping genes 18s and RPLP0 were used for normalizing gene expression from both HUVEC and HMVEC. All gene expression results were presented as normalized fold changes, calculated using 2^-∆∆C^_T_, compared to control (5.6 mM D-glucose).

### Data analysis

All experiments were carried out three times, except for hydrogen peroxide measurements, which had four replicates. Data analyses on all results were carried out using one-way analysis of variance (ANOVA) followed by Fisher’s LSD based post-hoc analysis. Comparisons were deemed significant for p-value ≤ 0.05. All quantified results were presented as mean ± S.E.M. (standard error of mean) in graphical representation.

## Results

### Gene expression of NOS and NOX family enzymes in hyperglycemia was upregulated in HMVEC but not in HUVEC

**Figure 1:**
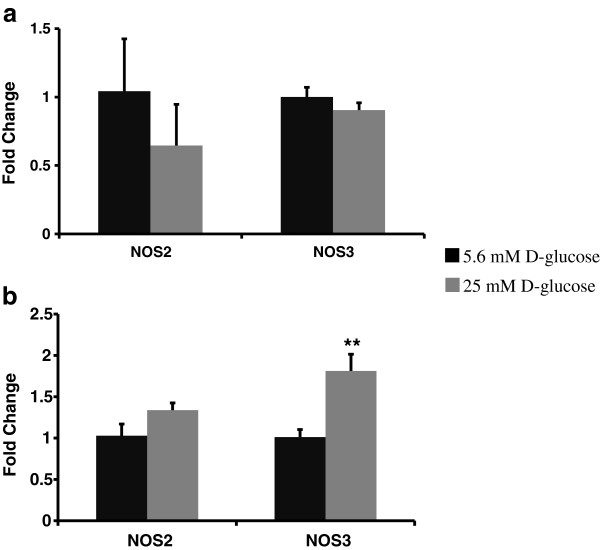
**Expression levels of NOS enzymes in HUVEC, and HMVEC after 24 hours of high glucose exposure.** Gene expression of NOS2 and NOS3 were measured following 24 hours of HG-M199 exposure to **(a)** HUVEC and **(b)** HMVEC. Fold changes were calculated by 2^-∆∆C^_T_ method using 18s and RPLP0 as housekeeping genes and cells grown in M199 media with 5.6 mM D-glucose as control. **p ≤ 0.05 significantly higher than control for respective cells type.

**Figure 2:**
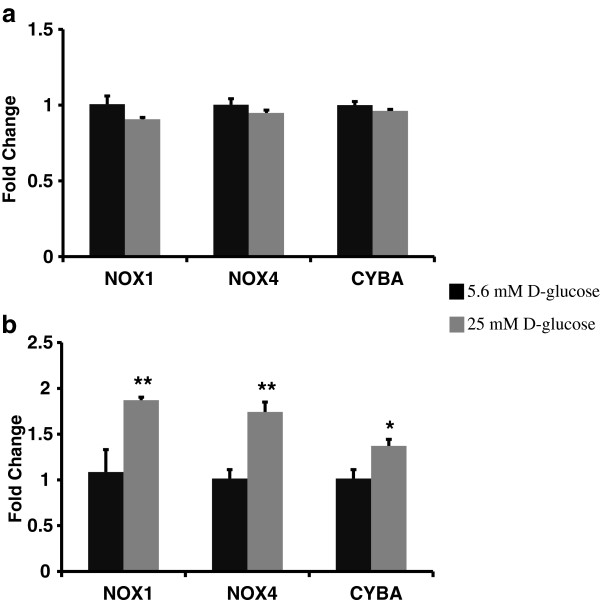
**Expression levels of NAD(P)H oxidase family enzymes in a) HUVEC, and) HMVEC cells after 24 hours of high glucose exposure.** Gene expression of NOX1, NOX4, and CYBA were measured using real-time PCR in both **(a)** HUVEC and **(b)** HMVEC. Fold changes were calculated by 2^-∆∆C^_T_ method using 18s and RPLP0 as housekeeping genes and cells grown in M199 media with 5.6 mM D-glucose as control. **p ≤ 0.05 significantly higher than control for HMVEC. *p ≤ 0.07 higher than control for HMVEC.

We exposed HUVEC and HMVEC to high glucose for 24 hrs. After 24 hours of exposure, RNA was isolated and synthesized to cDNA for real-time PCR based gene expression measurement. We measured the gene expression levels of NOS and NOX family enzyme, which are sources of NO and superoxide, respectively. As seen in Figure 1a, the gene expression levels of NOS2 and NOS3 were downregulated (though not statistically significant) in HUVEC after high glucose exposure. High glucose exposure to HMVEC increased NOS3 (p ≤ 0.05) and NOS2 (not significant, p = 0.15) expression (Figure 1b). Following high glucose exposure, the expression of NOX family genes did not change in HUVECs (Figure 2a) whereas the expression of NOX1 (p ≤ 0.05), NOX4 (p ≤ 0.05), and CYBA (p = 0.07) (Figure 2b) increased in HMVEC. Thus, high glucose induced significantly different response in NOS and NOX family enzyme in HMVEC and HUVEC.

### Hyperglycemia upregulated the gene expression of ROS and peroxide clearance enzymes in HMVEC whereas it downregulated in HUVEC

**Figure 3:**
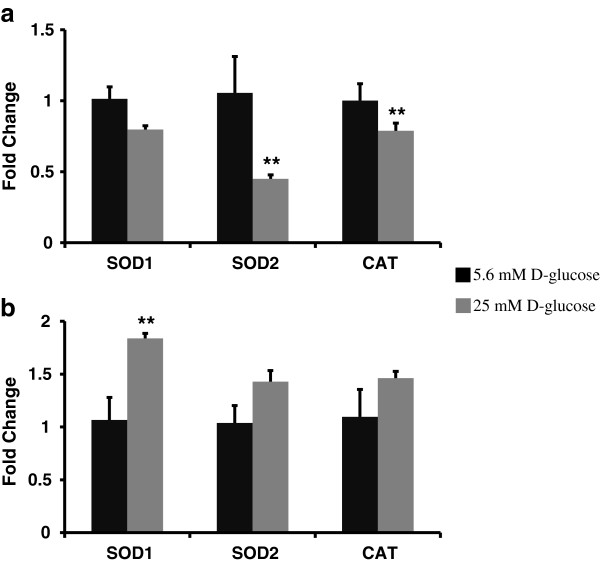
**Expression levels of ROS clearance enzymes in HUVEC, and HMVEC cells after 24 hours of high glucose exposure.** Gene expression of SOD1, SOD2, and CAT were measured in **(a)** HUVEC and **(b)** HMVEC following 24 hours of treatment with HG-M199. Fold changes were calculated by 2^-∆∆C^_T_ method using 18s and RPLP0 as housekeeping genes and cells grown in M199 media with 5.6 mM D-glucose as control. **p ≤ 0.05 significantly different than control for respective cells type.

**Figure 4:**
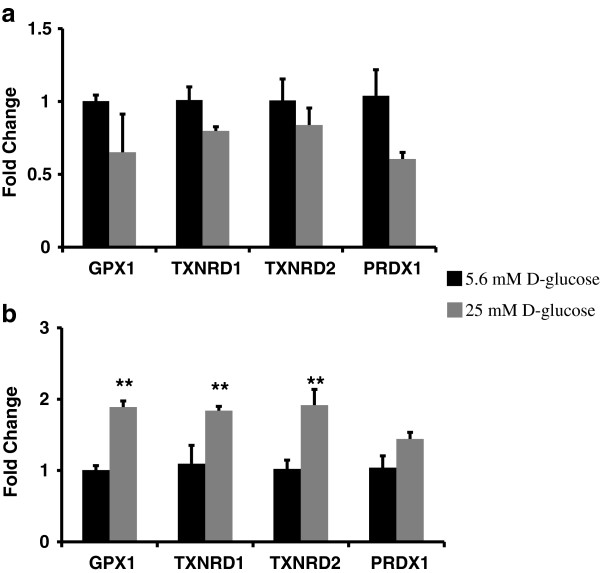
**Expression levels of peroxide clearance enzymes in HUVEC, and HMVEC cells after 24 hours of high glucose exposure.** Gene expression of GPX1, TXNRD1, TXNRD2, and PRDX1 were measured in **(a)** HUVEC and **(b)** HMVEC following 24 hours of exposure to HG-M199. Fold changes were calculated by 2^-∆∆C^_T_ method using 18s and RPLP0 as housekeeping genes and cells grown in M199 media with 5.6 mM D-glucose as control. **p ≤ 0.05 significantly higher than control for HMVEC.

After 24 hours of exposure to high glucose, gene expression levels of SOD1, SOD2, CAT, GPX1, TXNRD1, TXNRD2, and PRDX1 were measured to evaluate transcriptional changes leading to ROS and peroxide clearance in HUVEC and HMVEC. We observed an opposite transcription regulation of ROS and peroxide clearance enzymes in high glucose treated HUVEC and HMVEC. As seen in Figure 3a, the expression of SOD2 and CAT was significantly (p ≤ 0.05) downregulated in HUVEC post 24 hours of high glucose exposure. The expression of SOD1 (p = 0.16), GPX1 (p = 0.34), TXNRD1 (p = 0.11), TXNRD2 (p = 0.39) and PRDX1 (p = 0.11) was also downregulated (but not statistically significant) compared to control in HUVEC (Figure 4a). High glucose exposure upregulated gene expression of ROS and peroxide clearance enzymes in HMVEC. The expression of SOD1 (p ≤ 0.05), GPX1 (p ≤ 0.0005), TXNRD1 (p ≤ 0.05) and TXNRD2 (p ≤ 0.05) was significantly upregulated in high glucose treated HMVEC (Figures 3b and 4b). Moreover, the expression levels of SOD2 (p = 0.15), CAT (p = 0.21) and PRDX1 (p = 0.13) were also upregulated but not statistically different than the control (Figure 3b) in HMVEC. Thus, there was an overall upregulation in expression of ROS and peroxide clearance enzymes in HMVEC whereas in HUVEC their expression either downregulated or remained unchanged under hyperglycemia.

### Hyperglycemia upregulated oxidative stress activated transcription factor (NFE2L2) and UCP1 gene expressions in HMVEC but not in HUVEC

**Figure 5:**
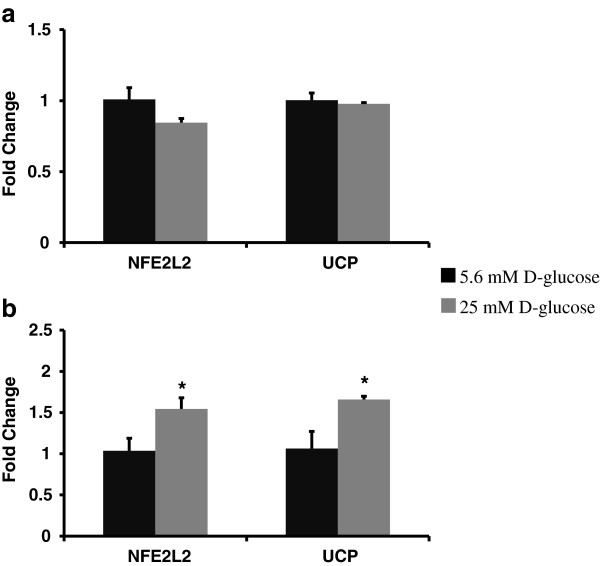
**Expression levels of oxidative stress responding transcription factor and uncoupling protein in HUVEC, and HMVEC cells after 24 hours of high glucose exposure.** Gene expression of NFE2L2 and UCP1 were measured in **(a)** HUVEC and **(b)** HMVEC following 24 hours of exposure to HG-M199. Fold changes were calculated by 2^-∆∆C^_T_ method using 18s and RPLP0 as housekeeping genes and cells grown in M199 media with 5.6 mM D-glucose as control. *p ≤ 0.06 higher than control for HMVEC.

We measured gene expression of NFE2L2 and UCP1 in high glucose treated HUVEC and HMVEC following 24 hour exposure. NFE2L2 translates to Nrf-2, which is a transcription factor for antioxidant enzymes, to maintain cellular redox state under oxidative stress condition. As seen in Figure 5, hyperglycemia induced a downregulation of NFE2L2 (p = 0.19) in HUVEC whereas it upregulated NFE2L2 expression in HMVEC (p = 0.06). Similarly, UCP1, which uncouples the electron transport chain from oxidative phosphorylation and thereby decreases mitochondrial ROS generation, expression was also differentially regulated in both endothelial cells. High glucose exposure induced no change in UCP1 in HUVEC (Figure 5a) whereas the expression of UCP1 was upregulated in HMVEC (p = 0.06, Figure 5b).

### Hyperglycemia increased intracellular O_2_‾ production, in both endothelial cells, that was accompanied with elevated H_2_O_2_ levels in HUVEC and not in HMVEC

**Figure 6:**
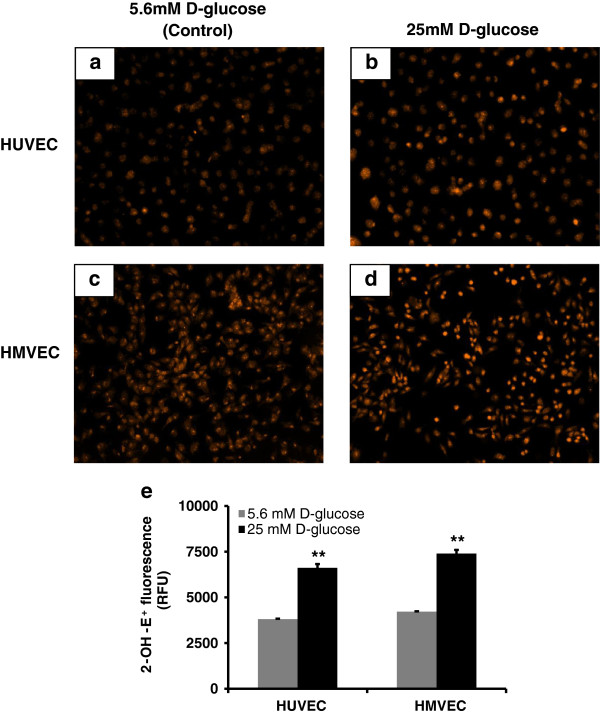
**O**_**2**_**‾ production in HUVEC and HMVEC cells following high glucose exposure for 24 hours.** Using DHE levels of O_2_‾ were measured in both HUVEC and HMVEC following 24 hours of high glucose treatment. Fluorescence imaging was used to capture DHE fluorescence in **(a-b)** HUVEC and **(c-d)** HMVEC. Fluorescence microplate reader based measurements were also performed to measure specific fluorescence intensity of 2-OH-E^+^ in **(e)** HUVEC and HMVEC treated with high glucose. For both types of DHE measurements, cells grown in 5.6 mM D-glucose were used as control. **p ≤ 0.01 significantly higher than control for respective cells type.

In order to understand how changes in these gene expression results in overall cellular ROS generation, we measured O_2_‾ and H_2_O_2_, levels in HUVEC and HMVEC. High glucose exposure induced a similar increase in O_2_‾ production in both HUVEC and HMVEC but H_2_O_2_ levels were regulated differently in both endothelial cells. Figure 6 shows the DHE measurement using fluorescence microscopy and a microplate reader. There was an increase in O_2_‾ production for both endothelial cells treated with high glucose for 24 hours as shown in DHE fluorescence images (Figure 6a-d). Furthermore, the specific product of DHE and O_2_‾ interaction, 2-OH-E^+^, significantly increased in both HUVEC and HMVEC (p ≤ 0.01) after high glucose exposure (Figure 6e).

**Figure 7:**
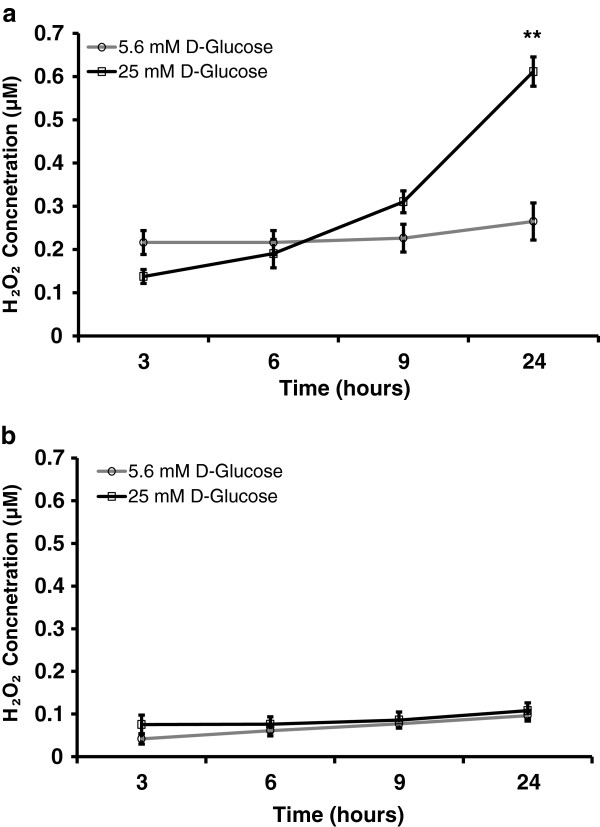
**H**_**2**_**O**_**2 **_**production profile in high glucose treated HUVEC and HMVEC.** Levels of H_2_O_2_ were measured in media supernatant of **(a)** HUVEC and **(b)** HMVEC treated with either 5.6 mM (control) or 25 mM D-glucose. Cells grown in media with 5.6 mM D-glucose were used as control. **p ≤ 0.006 significantly higher than control for respective cells type.

Figure 7 shows the H_2_O_2_ levels over 24 hours. Although there was no significant change in H_2_O_2_ levels at initial time points in HUVEC, H_2_O_2_ level at 24 hours was significantly higher than control (p ≤ 0.006) as seen in Figure 7a. On contrary, high glucose treatment did not change H_2_O_2_ level in HMVEC over 24 hours of exposure (Figure 7b). H_2_O_2_ production profiles were similar in controls for both cell types over 24 hours. Basal level of H_2_O_2_ in control was higher in HUVEC than in HMVEC. In addition, H_2_O_2_ production profiles were different and H_2_O_2_ levels were higher in hyperglycemic HUVEC compared to HMVEC.

### Hyperglycemia hyperpolarized mitochondrial membrane in HUVEC whereas it depolarized mitochondrial membrane in HMVEC

**Figure 8:**
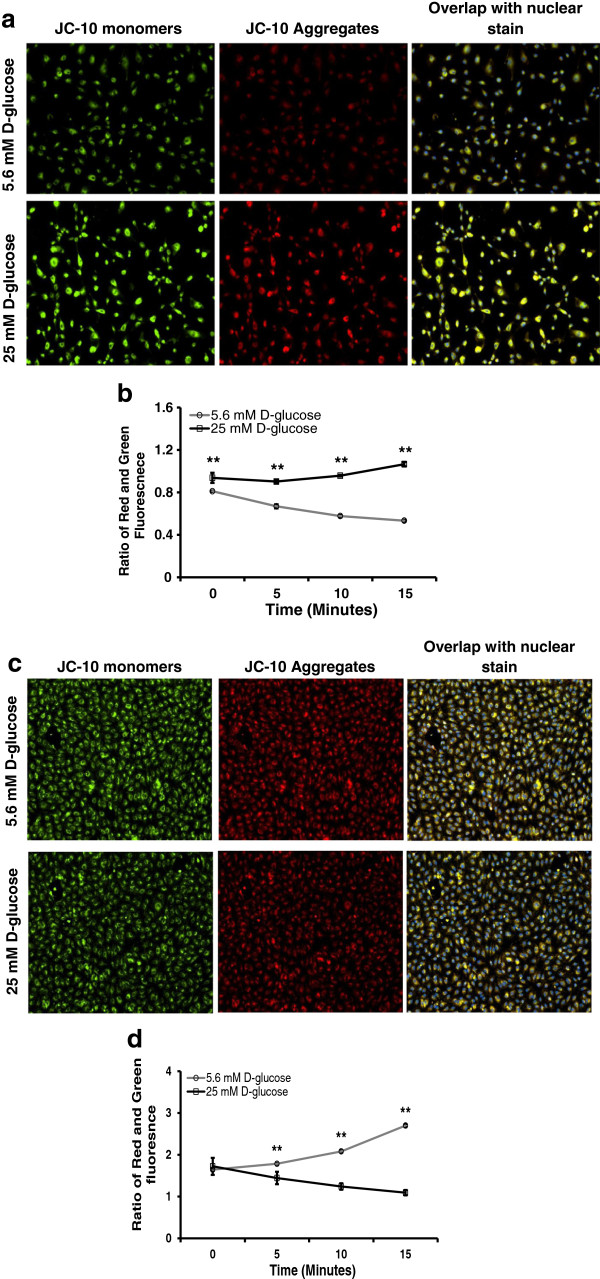
**Mitochondrial membrane polarization in HUVEC and HMVEC following high glucose treatment for 24 hours.** Using JC-10 mitochondrial membrane polarization was measured in both HUVEC and HMVEC after 24 hours of high glucose treatment. Mitochondrial membrane polarization of high glucose treated HUVEC was evaluated through measurement of JC-10 aggregates (Red) and monomers (Green) using fluorescence microscopy and microplate reader. **a)** Overlapped fluorescence images of red and green fluorescence intensities along with Hoescht-33342 based nuclear stain were used to show hyperpolarized mitochondrial membrane in high glucose treated HUVEC. **b)** Similarly, ratio of red and green fluorescence intensities were plotted to illustrate mitochondrial membrane hyperpolarization in high glucose treated HUVEC. **p ≤ 0.05 significantly higher than 5.6 mM D-glucose exposure treatment in HUVEC. **c)** Overlapped fluorescence images of red and green fluorescence intensities along with Hoescht-33342 based nuclear stain were used to show depolarized mitochondrial membrane in high glucose treated HMVEC. **d)** Ratio of red and green fluorescence intensities were plotted to show high glucose exposure-induced mitochondrial membrane depolarization in HMVEC. **p ≤ 0.05 significantly higher than 25 mM D-glucose exposure concentration in HMVEC.

To understand the effect of hyperglycemia-induced change in gene expressions and ROS level on mitochondrial activity, we measured mitochondrial membrane polarization in HUVEC and HMVEC over 24 hours using JC-10. As seen in Figure 8a, high glucose treated HUVEC had high red fluorescence because of JC-10 aggregation in mitochondria. In addition, the ratio of red to green fluorescence was significantly higher in high glucose treated HUVEC compared to control (p ≤ 0.05, Figure 8b). This indicated that mitochondrial membrane hyperpolarized after exposure to hyperglycemia in HUVEC. In HMVEC, high glucose exposure caused lower red fluorescence intensity (Figure 8c) and significantly lower red to green fluorescence ratio (p ≤ 0.05, Figure 8d) as compared to control. These changes in JC-10 fluorescence indicated that HMVEC mitochondrial membrane depolarized after exposure to hyperglycemia. Thus, high glucose exposure induced a complete opposite trend in mitochondrial membrane polarization in both HUVEC and HMVEC.

## Discussion

An understanding of hyperglycemia-induced endothelial dysfunction is important for therapeutic intervention of vascular complications in diabetes. In this study, we compared the hyperglycemia-related changes in primary human endothelial cells from both umbilical vein (HUVEC) and microvascular (HMVEC) origins. We investigated the regulation of hyperglycemia-induced functional changes and gene expression fingerprints for related enzymes in both ECs. We did not evaluate specific protein expression and activity levels as we observed various sources and enzyme networks were affected in this study; resulting in an overall contribution to O_2_‾ and H_2_O_2_ production. Moreover, limited correlation among protein translation, activity, and gene expression would not exclusively validate gene expression changes or vice versa [35-37].

Results from our study indicated that hyperglycemia induced consistent increase in O_2_‾ production in both ECs. However, there was a differential change in underlying gene expression profile, H_2_O_2_ and mitochondrial membrane polarization levels during high glucose exposure in both ECs. A significant up-regulation in gene expression levels of NOS and NOX family enzymes, SOD1, CAT, GPX1, TXNRD1, TXNRD2, NFE2L2 and UCP1 were observed in HMVEC under high glucose exposure. On the contrary, HUVEC showed a down-regulation in SOD2 and CAT enzymes along with elevated H_2_O_2_ levels and hyperpolarized mitochondria.

### Hyperglycemia-induced differential NOS enzyme regulation in HUVEC and HMVEC

Hyperglycemia results in endothelial dysfunction that may be a consequence of or cause of change in NOS expression and activity levels. Variable changes in NOS expression have been reported in the literature for hyperglycemic-endothelial cells in cell cultures, animal models and humans [38]. We showed a non-significant downregulation and upregulation of NOSs expression in HUVEC and HMVEC, respectively. The expression levels of NOS3 have been have been reported to be up, down and no change in HUVEC [32,39]. Following 24 hours of high glucose exposure to HUVEC, we showed a non-significant downregulation in NOS3, which was similar to unchanged NOS3 levels reported by Zielonka et al. [32]. In HMVEC, we showed that the hyperglycemia induced an upregulation of NOS3 and NOS2 gene expression levels similar to previous study [40]. This differential NOS expression in HUVEC and HMVEC suggests a vasculature specific vasoregulatory-gene expression regulation in hyperglycemia.

### Differential NAD(P)H oxidase family enzyme regulated a consistent O_2_‾ production in high glucose treated HUVEC and HMVEC

Elevated O_2_‾ levels serves as a hallmark to hyperglycemia mediated oxidative stress and vascular dysfunction [6,29,32]. We showed that DHE based measurement revealed increased O_2_‾ levels in both endothelial cells. NAD(P)H oxidase family enzymes play a pivotal role in O_2_‾ production in variety of endothelial cells during hyperglycemia [41]. Their ability to regulate O_2_‾ production has been linked with their translocation to plasma membrane to co-localize with other enzyme units and increased activation under hyperglycemic condition [42-44]. Thus, even without any change in gene expression levels, as seen in our results, NAD(P)H oxidase family enzymes can regulate O_2_‾ production in HUVEC. Additionally, eNOS uncoupling and mitochondrial ROS can contribute to increased cellular O_2_‾, as seen in results [14,45].

In our study, hyperpolarized mitochondrial membrane suggested the involvement of mitochondria in O_2_‾ production in HUVEC. However, a depolarized mitochondrial membrane in hyperglycemic-HMVEC indicated involvement of non-mitochondrial O_2_‾ production sources. Though the specific details of O_2_‾ production sources in HMVEC are not available in the literature, our results suggested an increased involvement of NAD(P)H oxidase family enzyme (CYBA (non-significant), NOX1, and NOX4) in hyperglycemic HMVEC.

Along with increased involvement of O_2_‾ production sources, we also showed downregulated SOD1 (non-significant) and SOD2 expression in HUVEC. Downregulated gene expressions of SODs were suggestive to reduced O_2_‾ clearance in high glucose treated HUVEC. In HMVEC, gene expression of SOD1 and SOD2 (non-significant) simultaneously increased with O_2_‾ levels, and CYBA (non-significant), NOX1, and NOX4 expression during hyperglycemia. Thus, hyperglycemia can induce regulation in gene expression of pro- and anti-oxidant sources resulting in increased O_2_‾ levels through a complex interplay amongst them. Previous studies from our group have analyzed the complex interactions amongst oxidative stress and SOD [46,47]. Moreover, enzymatic activity regulation in pro- and anti-oxidant sources could also influenced increased O_2_‾ levels as seen in high glucose treated HMVEC [36,37].

### Interplay among mitochondrial membrane polarization and peroxidase clearance enzyme yielded to differential H_2_O_2_ in both endothelial cells

Excess production of H_2_O_2_ through rapid O_2_‾ dismutation also contributes to hyperglycemia-dependent oxidative stress. H_2_O_2_ is one of the stable forms of free radicals during oxidative stress. However, H_2_O_2_ levels in hyperglycemic condition are variably reported in literature and hence its role in vasoregulatory activity remains unclear [38]. Consistent with the literature [48,49], we showed increased levels of H_2_O_2_ in HUVEC. However, no-change was observed in H_2_O_2_ levels of HMVEC during high glucose exposure. Furthermore, we observed higher basal level H_2_O_2_ in HUVEC compared to HMVEC in controls. Under physiological condition, H_2_O_2_ promotes NO production and maintains cell growth [50,51]. Basal H_2_O_2_ levels are not well characterized across vasculature but our results suggests a possibility of higher basal production of H_2_O_2_ in macrovessel (HUVEC) compared to microvessel (HMVEC). The H_2_O_2_ levels are dependent on O_2_‾ dismutation reaction, peroxide clearance, NAD(P)H oxidase activation and crosstalk with mitochondria, and mitochondrial hyperpolarization.

In HUVEC, downregulated SOD2 and CAT gene expression, similar as observed by Felice et al. [52], and their reduced activity under high glucose exposure, as shown by Zhang et al. [42], suggested decreased O_2_‾ and H_2_O_2_ removal in HUVEC. As a result increased H_2_O_2_ level prevailed along with higher O_2_‾ level in HUVEC following high glucose exposure. Moreover, hyperpolarized mitochondrial membrane, associated with increased H_2_O_2_ production, in HUVEC contributed to overall H_2_O_2_ levels in hyperglycemia [53]. Additionally, we observed downregulated (non-significant) gene expression levels of GPX1, TXNRD1, TXNRD2, PRDX1 and NFE2L2 in HUVEC, which plays a pivotal role in peroxidase clearance and promoting an anti-oxidative environment. Hyperglycemia-induced unaltered GPX1 was similar as reported by Felice et al. [52]. Not much detail is available in literature for hyperglycemia-induced gene expression of NFE2L2, TXNRD1, TXNRD2 and PRDX1 in HUVEC. In our results for HUVEC, hyperglycemia-induced regulation of these peroxidase clearance genes to pro-oxidative cellular environment and a reduced peroxide clearance.

Interestingly in contrast to HUVEC, the gene expression of ROS clearance enzymes, SOD1, GPX1, TXNRD1, TXNRD2, and PRDX1 were upregulated in HMVEC under hyperglycemia. ROS clearance transcription factor, NFE2L2 was also upregulated (though non-significant, p = 0.06) under similar conditions. Upregulated SOD1 expression level suggests an increased O_2_‾ processing to H_2_O_2_ in high glucose treated HMVEC. But simultaneous upregulation of GPX1, TXNRD1, TXNRD2, PRDX1 and NFE2L2 expression indicated a balanced increase in H_2_O_2_ clearance in high glucose treated HMVEC. Along with simultaneous expression of SOD1 and peroxide clearance enzymes, we observed no increase in H_2_O_2_ level indicating a predominant involvement of peroxide clearance enzymes in HMVEC under high glucose exposure. Additionally our results also showed depolarized mitochondrial membrane along with increased UCP1 gene expression, which has been shown to downplay mitochondrial H_2_O_2_ production in HMVEC [53]. Since there was not enough evidence in literature, we used our results from experiments using HUVEC as a reference to report these differential findings from HMVEC for the first time.

### Differential regulation of mitochondrial membrane polarization through UCP1 expression

Uncoupling proteins (UCPs) are functionally linked to shunt electron transport chain and reduce mitochondrial membrane polarization in endothelial cells. Several studies explored this trait of UCPs by overexpressing UCP1, UCP2, and UCP3 to ameliorate mitochondrial hyperpolarization and hence to reduce ROS production in endothelial cells [45,54]. However, it was believed that UCP1 expression was exclusive to brown adipose tissue, but increasing number of recent studies have reported presence of UCP1 transcription in other tissues and specifically in endothelial cells from bovine and human retina [55]. In our results, we reported the presence of UCP1 transcription in both HUVEC and HMVEC. We also reported that hyperglycemia did not change UCP1 gene expression in HUVEC but upregulated it in HMVEC (though non-significant, p = 0.06). As observed by Nishikawa et al. [45,54], increased UCP1 gene expression observed in HMVEC can be linked to depolarized mitochondrial membrane and unchanged H_2_O_2_ production [53]. As the current state of literature is not clear on regulation and role of all UCPs in endothelial cells, it will be important to characterize gene expression of other UCPs in future studies.

### Hyperglycemia-induced differential epigenetic regulation in ECs from different vasculature

Emerged concepts in vascular metabolic memory have explained limited efficacies of antihyperglycemic treatment and its late effect in type 2 diabetes [56]. The theories justifying such involvement of vascular metabolic memory have implicated non-reversible epigenetic regulation [57]. As observed in our results, 24 hours of hyperglycemia-induced differentially regulated gene expression in HUVEC and HMVEC. In specific, high occurrences of gene expression changes in HMVEC in our study provided the evidence for the hypothesis in vascular metabolic memory that suggests microvasculature precedes macrovasculature in epigenetic regulation forming vascular metabolic memory [58]. Such differential regulation, even from short exposure, may explain non-uniform epigenetic regulation across vasculature. Further research will be of importance to understand the involvement of vasculature specific pathologies and the role of vascular metabolic memory in endothelial dysfunction.

## Conclusion

In conclusion, we studied the pro and anti-oxidant sources contributions under hyperglycemic endothelial cell oxidative stress. Hyperglycemia induced a pro-oxidative environment in both HUVEC and HMVEC through increased O_2_‾ production. We demonstrated that ECs have differential involvement of oxidative stress regulating mechanisms from NOS enzymes, NAD(P)H oxidase enzymes, ROS clearance enzymes and antioxidant gene expression in hyperglycemia that results in significantly different outcome of mitochondrial membrane polarization and H_2_O_2_ level. Such interplay among key enzyme gene expression, mitochondrial membrane polarization and O_2_‾ and H_2_O_2_ level regulation indicated the complexity of processes that regulates vasculature specific endothelial behavior in hyperglycemia. High occurrences of gene expression changes in HMVEC in this study supports the hypothesis that microvasculature precedes macrovasculature in epigenetic regulation forming vascular metabolic memory. Identifying genomic phenotype and corresponding functional changes in hyperglycemic endothelial dysfunction will provide a suitable systems biology approach for understanding underlying mechanisms and possible effective therapeutic intervention.

## Abbreviations

O2‾: Superoxide; H2O2: Hydrogen peroxide; ROS: Reactive oxygen species; DHE: Dihydroethidium; NO: Nitric oxide; EC: Endothelial cell; eNOS: Constitutive nitric oxide synthase; SOD: Superoxide dismutase; NOX4: NAD(P)H oxidase-4; HUVEC: Human umbilical vein endothelial cell; HAEC: Human aortic endothelial cell, HREC, Human retinal endothelial cell; HGEC: Human glomerular endothelial cells; HMEC-1: Human microvascular endothelial cells; HMVEC: Human dermal microvasculature endothelial cell; SOD1: Superoxide dismutase 1; SOD2: Superoxide dismutase 2; CAT: Catalase; PRDX1: Peroxiredoxin; GPX1: Glutathione peroxidase 1; TXNRD1: Thioredoxin reductase 1; TXNRD2: Thioredoxin reductase 1; NFE2L2: Oxidative stress activated transcription factor, Nrf-2; UCP1: Uncoupling protein 1; HG-M199: 25mM D-glucose added M199; DHE: Dihydroethidium; 2-OH-E+: 2-hydroxyethidium; Ex: Excitation; Em: Emission; ANOVA: Analysis of variance; S.E.M.: Standard error of mean; UCPs: Uncoupling proteins.

## Competing interests

No potential conflicts of interest relevant to this article were reported.

## Authors’ contributions

HP designed study, researched gene expression, H_2_O_2_ and mitochondrial membrane polarization measurement data, performed statistical analysis, performed literature search and drafted manuscript. JC researched O_2_‾ production results. HP and JC are the guarantor of this work and takes responsibility for the integrity of the data and accuracy of the data analysis. KD reviewed manuscript. MK reviewed and edited manuscript. All authors approved the final manuscript for publication.
